# Analysis of Facial Occlusion Challenge in Thermal Images for Human Affective State Recognition

**DOI:** 10.3390/s23073513

**Published:** 2023-03-27

**Authors:** Mustafa Al Qudah, Ahmad Mohamed, Syaheerah Lutfi

**Affiliations:** 1 School of Computer Sciences, Universiti Sains Malaysia, Gelugor 11800, Penang, Malaysia; 2 Department of Computer Science, College of Science and Humanities in Al-Sulail, Prince Sattam bin Abdulaziz University, Kharj 16278, Saudi Arabia

**Keywords:** affects, emotion, recognition, thermal, occlusion, eyeglass

## Abstract

Several studies have been conducted using both visual and thermal facial images to identify human affective states. Despite the advantages of thermal facial images in recognizing spontaneous human affects, few studies have focused on facial occlusion challenges in thermal images, particularly eyeglasses and facial hair occlusion. As a result, three classification models are proposed in this paper to address the problem of thermal occlusion in facial images, with six basic spontaneous emotions being classified. The first proposed model in this paper is based on six main facial regions, including the forehead, tip of the nose, cheeks, mouth, and chin. The second model deconstructs the six main facial regions into multiple subregions to investigate the efficacy of subregions in recognizing the human affective state. The third proposed model in this paper uses selected facial subregions, free of eyeglasses and facial hair (beard, mustaches). Nine statistical features on apex and onset thermal images are implemented. Furthermore, four feature selection techniques with two classification algorithms are proposed for a further investigation. According to the comparative analysis presented in this paper, the results obtained from the three proposed modalities were promising and comparable to those of other studies.

## 1. Introduction

The human affective state is an important factor that greatly influences our lifestyle, including our thoughts, activities, thinking, focus, and our problem-solving and decision-making abilities. Thus, affective state recognition is of considerable interest to researchers. Affect, mood, and emotion are concepts included in the domain of affective computing. Affect is a general term for a range of feelings that a person can experience. It includes emotions, which are intense feelings that can be directed at a source and are usually short-lived, and moods, which are longer-lasting, less intense, and may not require a specific stimulus [1,2].

Researchers have recently been looking into techniques to recognize human emotions and are applying them to a variety of fields. This includes human–computer interaction to facilitate communication between humans and computers. Human–robot interaction involves understanding how people behave and feel, as this helps the robot to interact with people in an appropriate manner [3]. Human emotion techniques have been used in security applications to identify people who are able to mask their emotions, often referred to as having a “poker face” [4], and in deception detection to detect when someone has not been truthful or accurate in their statements [5,6]. They have also been used for medical applications, including people with autism disorder who may not be able to use their body language, facial expressions, or spoken language to show how they feel. Therefore, they require assistance to help with their specific needs in order to understand and express their emotions [7]. Other medical applications include sleep apnea, which causes a person’s breathing to become shallow or to temporarily stop when they are sleeping [8].

Human affect can be identified by observing the different characteristics of a person, such as their facial expressions, the way they speak, their gestures, the way they stand (their pose), and the direction they are looking. More importantly, previous studies have mentioned that emotional messages are conveyed through facial expressions 55% of the time and through spoken words 7% of the time, while the remaining message is expressed by the manner in which words are spoken, known as paralanguage [9]. Numerous approaches, based on contactless and noninvasive methods, have been applied in emotion recognition and include speech emotion recognition, gesture emotion recognition, and facial emotion recognition, while other studies have concentrated on physiological signals, such as human temperature, cardiac signals, respiration rate, and sweat gland activities.

The human face is often used as a source of information because observing the face is a noninvasive way to collect data. This means that it does not require physical contact or the use of medical instruments. Therefore, it is used in different areas of research, and many studies have been conducted using visible facial images. Despite productive results being reported in the facial emotion recognition literatures, limitations to technique, such as visible images being sensitive to illumination variation [10,11,12,13] and diversity in skin color, facial shape, and facial texture, could all contribute to differences in accuracy for the recognition of emotions via facial features. Additionally, different ethical backgrounds and cultural differences could also have an effect, meaning that what is considered happy or sad may be understood differently by different people [10,14]. Furthermore, the discrimination between spontaneous and deceptive emotions is challenging [13]. Therefore, implementing visual-based imaging could contribute to reducing accuracy of affective state recognition [15,16,17].

Recently, researchers have focused on the thermal imaging approach for affective state recognition because of its ability to reveal a heat pattern consisting of fluctuations in skin temperature caused by involuntary blood circulation that correlates to a particular human expression. Due to the involuntary nature of blood flow, actual emotions may be discernible in the thermal signature [6]. Moreover, the arousal of emotions can cause subtle temperature changes in different regions of the face, such as the forehead, periorbital, supraorbital, tip of the nose, lips, and maxillary [18]. Thermal imaging could be a potential solution to overcoming the illumination variation problem and facilitating the recognition of spontaneous emotion rather than visible images. Although thermal imagery has many tangible advantages, this technique has a variety of drawbacks. One of the limitations is that eyeglasses appear opaque in thermal images and thus decrease the thermal sensitivity of the informative facial regions, such as the inner eye, supraorbital, and periorbital locations [11,19,20,21]. Moreover, facial hair is another obstacle in thermal imaging which affects the accuracy of temperature measurements [21,22,23]. A few studies have focused on facial occlusion in affective state recognition. For example, Wang et al. [19] reduced the effects of spectacles by using a thermal temperature pattern; for each frame, the study calculated the mean temperatures of the ambient points, then calculated the temperature differences between each frame and its preceding frame, and finally updated the temperatures of all points for each frame by subtracting the temperature differences. Basu et al. [23] applied a median filter and CLAHE and selected six facial regions, including the forehead, left and right eyes, left and right cheeks, nose, and mouth, proposing Hu’s seven moment invariant method to extract features. Nguyen et al. [11] proposed an improved ROI extraction process to overcome eyeglass opacity. Nguyen et al. [20] proposed the fusion of thermal and visual images to overcome the eyeglass occlusion problem.

However, the goal of this study was to investigate more efficient facial subregions in thermal images to overcome eyeglass and facial hair occlusion and to enhance facial affective state recognition. More importantly, the proposed solution in this study comprises three models, with each having its own objective. The first proposed model applies six main ROIs. The eye region is excluded, and other facial areas are employed, including the forehead, tip of the nose, cheeks, mouth, and chin. Moreover, the goal of this model is to recognize the human affective state without implementing important eye ROIs, contrary to previous studies, which means that abandoning eye ROIs could lead to overcoming the eyeglass occlusion challenge. The second proposed model in this study divided the 6 main ROIs into 27 sub-ROIs; the goal of this model is to explore the efficiency of facial sub-ROIs in recognition human affects rather than using the main ROIs, which could have partial occlusion. The third proposed model in this study conducted affective state recognition based on 11 selective sub-ROIs. The selected ROIs located on facial patches with the free existence of facial hair, such as a beard, mustaches, and hair bangs. Therefore, the goal of this model is the recognition of the human affective state even when wearing eyeglasses and with the facial hair occlusion.

## 2. Previous Studies

This section presents a brief overview of previous studies related to human affective state recognition based on thermal imaging and discusses the main stages, such as dataset collection, preprocessing, facial regions of interest (ROIs), feature extraction methods, and classification algorithms.

### 2.1. Thermal Dataset

The sophistication of thermal sensors has encouraged researchers to use thermal images to recognize human affects. According to previous studies, the dataset collection stage is an important process; thus, several studies constructed their own dataset [22,24,25,26], while others used already-published databases, such as KTFE or USTC-NVIE [11,27,28].

Depending on the emotion type, thermal datasets can be classified as posed or spontaneous [29]. Individuals in the posed dataset were asked to express a variety of emotions in order to identify their posed emotional state. This means that this type of emotion does not accurately represent the affective state. In contrast, participants were exposed to stimuli and were unaware that they were being recorded to elicit their spontaneous affective state while their actions were being recorded. As a result, creating spontaneous databases is a difficult process [30].

### 2.2. Preprocessing

In the preprocessing stage, literatures have proposed various methods to enhance thermal images and localize faces; for instance, HOG and SVM were used by Kopaczka et al. [28] to extract faces from thermal images. In order to identify the region of the initial frame and calculate the head motion, Liu and Yin [31] proposed a model for face identification which comprises a combination of trees with the shard pools of parts drawn from [32]. Otsu thresholding is an approach used in image processing to transform a grayscale image into a black and white image. Wang et al. [13] applied the Otsu thresholding method to create a binary image, and then they analyzed the vertical and horizontal curves of that image to determine the gradient with the highest value; this was used to identify the facial boundary. Latif et al. [33] conducted contrast limited histogram equalization to enhance image contrast. Moreover, to detect the facial region, Mohd et al. [10] used a computer vision technique known as the Viola–Jones boosting algorithm combined with a series of Haar-like features to identify facial regions within a thermal image. Wan et al. [19] proposed a method to help identify the face in an image. The technique uses temperature space, which means that it looks at how hot or cold different parts of an image are. This allows the algorithm to tell which parts are the face and which parts are the background. For face detection, Goulart et al. [24] designed a process to detect a face in a thermal image. This process used three different kinds of filters: median filters, gaussian filters, and a binary filter.

### 2.3. Region of Interests (ROIs)

Human affects contribute significantly to temperature differences in facial regions. More specifically, the sympathetic nervous system (SNS) responds to human affects by controlling a variety of physiological signals, such as increasing blood flow, which then causes an increase in body temperature, propagated to the surface of the face. Consequently, thermal imaging could detect minor differences in facial temperature [33]. Moreover, variations in facial temperatures are caused by contractions in the facial action unit during human affects [34]. Numerous facial regions have been focused on by previous studies to measure the human affective state, including the forehead, tip of the nose, eyes, mouth, cheeks, and chin [6,10,24,31,33,34]. Differences in temperature values according to a specific emotion type have been reported in numerous studies; for example, Cruz-Albarran et al. [35] mentioned that the temperature of right and left cheeks increased when showing the emotion of being sad or disgusted, whereas the temperature of the maxillary and nose decreased when showing the sad or disgusted emotion. Ioannou et al. [36] showed that forehead temperature decreased when expressing sad or fear emotions and that it increased when showing the anger emotion. Moreover, the study of Jian et al. [37] reported that there is a positive correlation in the cheek and eye regions related to human emotions. Rooj et al. [6] selected facial ROIs such as cheeks, forehead, nose, and maxillary. Kumar et al. [38] proposed facial landmarks with the DenseNet model for facial ROI localization and extraction. Saha et al. [22] proposed the DFTA model to propose eight small facial patches, which contain important information which contributes to differentiating between emotion classes.

### 2.4. Feature Extraction

The type of selected features plays an important role in classification accuracy; thus, previous studies have used various types of features in their work. For example, statistical features including mean, variance, covariance, median, minimum, maximum, and histogram statistical features [13,20,23,24,25,28,34,39]. Other feature types which have been used are GLCM features [13,33,40], HOG features (HOG) [28], and LBP features [22,33,34]. Some studies in the literature have also used features from deep learning methods, such as transfer learning from Alex Net [41] and convolutional sparse coding [6].

### 2.5. Classification

Numerous classification algorithms have been utilized in human affective recognition. For example, the support victor machine algorithm (SVM) has been widely used in previous studies [13,28,31,33,42]. The local discriminant analysis classifier (LDA) [24,25,26], deep Boltzmann machine (DBN) [13], and deep learning classification algorithms [2,38,41,43,44] have also been utilized.

## 3. Proposed Methodology

**Figure 1:**
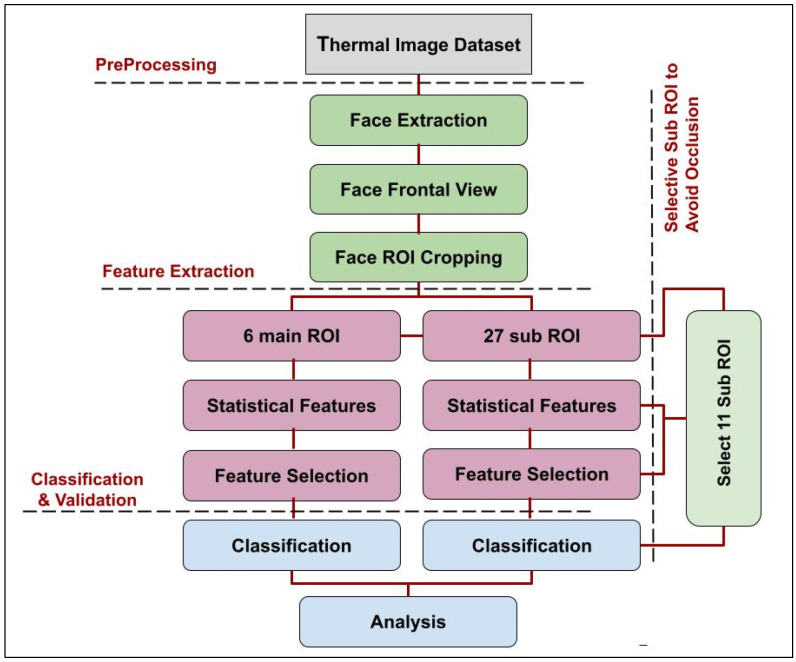
Stages of facial affective state recognition using thermal images with occlusion.

The proposed method in this study consists of three classification models, each with a different number of ROIs. The goal of implementing three models with different numbers of ROIs is to encompass all situations of occlusion presence and explore the efficiency of sub-ROIs in classifying human affects. For example, if a facial image only contains eyeglass occlusion, model one can handle this because it excludes eyes patches from the ROIs. Furthermore, model three can be used to avoid occlusion when facial images include both eyeglasses and facial hair, such as beards or mustaches. The main stages for proposed models are demonstrated in Figure 1. The first stage is the preprocessing of facial images prior to classification; facial images were extracted from their backgrounds and a frontal view process was used to ensure that all the faces had the same correlation due to spontaneous emotions, which are usually accompanied by head movements. Then, 6 main ROIs were cropped from facial patches and subdivided into 27 sub-ROIs. In the proposed models, the following ROIs are used: the first model has 6 main ROIs, the second model has 27 sub-ROIs, and the third model has 11 selective sub-ROIs. The next stage implemented nine statistical features for three models and four types of feature selection algorithms were applied, such as principal component analysis (PCA), analysis of variance (ANOVA), neighborhood components analysis (NCA), and naive Bayes (NB). The last stage focused on the classification of an affective state based on SVM and MLP for the three proposed models.

### 3.1. Dataset Selection

This study selected a dataset based on numerous factors. The first one is the recognition of a spontaneous affective state. The second requirement is that onset (beginning of emotion intensity) and apex (maximum emotion intensity) frames should be available for each individual in order to compute statistical features between two images. The third factor focused for the current study is facial occlusion, which includes eyeglasses and facial hair. As a result, the USTC-NVIE [27] database was selected because it satisfied the previous factors. The database provides six basic spontaneous emotions, which are happy, disgust, fear, surprise, anger, and sad. Furthermore, the database applied an evaluation process to ensure the intensity of an emotion for each class through five experienced evaluators. Based on their evaluation report, the study conducted an outlier process to select subjects with a higher emotion intensity, and after detecting the outliers, the number of instances employed for each class was as follows: happy: 99 subjects, disgust: 81 subjects, fear: 55 subjects, surprise: 65 subjects, anger: 56 subjects, and sad: 73 subjects.

### 3.2. Preprocessing Stage

**Figure 2:**
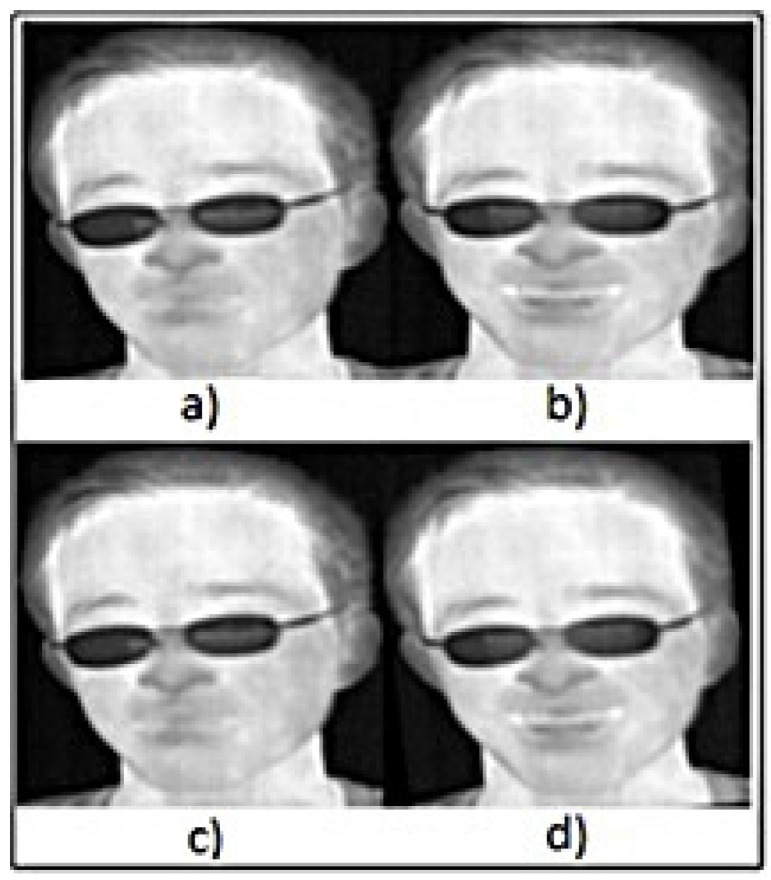
Image registration process: (**a**,**b**) represents onset and apex thermal images before similarity transformation, respectively; (**c**,**d**) represents onset and apex images after similarity transformation, respectively.

As demonstrated in Figure 1, facial extraction is the first step in the preprocessing stage. Several approaches have been accomplished in previous studies to extract facial regions, for example, HOG with SVM [45], face detection based on eye coordination and template matching [46], and the Viola–Jones algorithm [33]. The current study applied Goulart et al.’s [24] approach to extract thermal faces by using median and Gaussian filters with further preprocessing stages. More importantly, both onset and apex images have been selected to extract statistical features between them. Therefore, the facial extraction process was applied on both images, which are related to the same subject. Spontaneous emotions were accompanied with facial movements [47]. Therefore, to preserve the same coordination and frontal view for onset and apex images, this study applied image registration by conducting a similarity transformation. The images in Figure 2a,b are the onset and apex images before the similarity transformation, and those in Figure 2c,d are the onset and apex images after conducting a similarity transformation.

### 3.3. Selected ROIs

**Figure 3:**
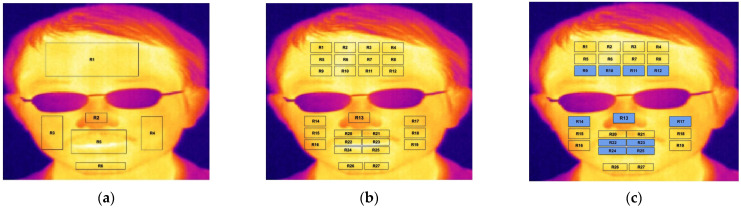
Selection of ROIs for three proposed classification models: (**a**) represents 6 main ROIs, (**b**) represents 27 sub-ROIs decomposed from 6 main ROIs, (**c**) represents selective 11 sub-ROIs from 27 sub-ROIs.

Despite the fact that the aforementioned studies from the literature have focused on several facial regions, such as the forehead, tip of the nose, eyes, mouth, cheeks, and chin [44,48,49], very few previous studies tackled the challenge of manipulating facial occlusion. Therefore, the current study selected facial images with eyeglass occlusion and excluded the important eye region from the selected ROIs. Moreover, this study utilized three classification models to explore the efficiency of ROIs when thermal face comprised occlusion. For example, the first classification model employed six main facial ROIs, including the forehead, tip of the nose, cheeks, mouth, and chin. The second classification model focused on decomposing the previous six main ROIs into 27 sub-ROIs to explore the efficiency of sub-ROIs in the recognition of human affects. The third proposed classification model in this study selected 11 sub-ROIs from 27 sub-ROIs; the selection process conducted based on the criteria of each sub-ROI should be free of facial hair, as the goal of this study is to tackle the challenge of manipulating facial occlusion. Figure 3 demonstrates the proposed three types of selective ROIs for each classification model. The selection of facial ROIs for three proposed classification models in this study is illustrated in the following steps:

- Classification based on six main ROIs: forehead, tip of the nose, left cheek, right cheek, mouth, and chin.
- Classification based on subdividing 6 main ROIs into 27 sub-ROIs as the following:
  1. Subdivide forehead main ROI into 12 sub-ROIs.
  2. Use tip of the nose ROI without subdividing.
  3. Subdivide left cheek main ROI into three sub-ROIs.
  4. Subdivide right cheek main ROI into three sub-ROIs.
  5. Subdivide mouth main ROI into six sub-ROIs.
  6. Subdivide chin main ROI into two sub-ROIs.
- Selected 11 sub-ROIs from 27 sub-ROIs, as demonstrated by the blue color in Figure 3c, labeled as: R9, R10, R11, R12, R13, R14, R17, R22, R23, R24, and R25.
- ROIs extraction in this research accomplished by manually selecting the bounding box on the main facial ROI for the apex image and the coordination of the bounding box applied on onset image to preserve the same ROIs coordination for both the onset and apex images. However, some previous studies have been conducted using the automatic ROI extraction method, but this technique exceeds the scope of the current study.

### 3.4. Feature Extraction

Before the feature extraction process, the facial regions were converted to temperature values using the equation from [50]. Therefore, the features were calculated based on temperature values instead of gray level intensity values. However, this research proposed nine statistical features to explore the variation in emotion intensity between apex and onset images for each emotion class, as demonstrated in the following:

Equation (1): the mean value of the temperature points ($\bar{X})$ in the apex image. $f_{1}$ represents feature one.

(1) $$f_{1}=\bar{X}=\frac{1}{l.m}\;\overset{l}{\underset{i=1}{\sum }}\overset{m}{\underset{j=1}{\sum }}X_{i\;j}$$

Equation (2): the mean value of the temperature differences ($\bar{xd}$) between the onset and apex images. Variable $f_{2}$ represents feature two.

(2) $$f_{2}=\bar{xd}=\frac{1}{l.m}\overset{l}{\underset{i=1}{\sum }}\overset{m}{\underset{j=1}{\sum }}Xd_{i,j}$$

Equation (3): variation in the temperature points in the apex image. Variable $f_{3}$ represents feature three.

(3) $$f_{3}=Variance\left(x\right)$$

Equation (4): variation in the temperature differences (Xd) between the onset and apex images. Variable $f_{4}$ represents feature four.

(4) $$f_{4}=Variance\;\left(Xd\right)$$

Equation (5): the maximum temperature value obtained from the apex image. Variable $f_{5}$ represents feature five.

(5) $$f_{5}=Max\left(X\right)$$

Equation (6): the minimum temperature value obtained from the apex image. Variable $f_{6}$ represents feature six.

(6) $$f_{6}=Min\left(X\right)$$

Equation (7): the mean of the maximum and minimum temperature values obtained from the apex image. Variable $f_{7}$ represents feature seven.

(7) $$f_{7}=\mathrm{Mean}\;(f_{5}+f_{6})$$

Equation (8): the median of the temperature values for the apex image. Variable $f_{8}$ represents feature eight.

(8) $$f_{8}=Median\left(X\right)$$

Equation (9): the median of the temperature differences (Xd) between the onset and apex images. Variable $f_{9}$ represents feature nine.

(9) $$f_{9}=Median\left(Xd\right)$$

After conducting statistical features, the number of features for the first, second, and third classification models in this research are 54, 243, and 99 features, respectively. Consequently, for an efficient classification, the feature reduction technique is required due to the large number of features. Four types of feature selection methods were proposed to explore the more efficient features. The selective feature reduction methods include principal component analysis (PCA), analysis of variance (ANOVA), neighborhood components analysis (NCA), and feature selection based on the naïve Bayes (NB) algorithm. After the feature selection algorithms arrange the features according to their importance in the classification, the first 50 features were selected from the ANOVA and PCA algorithms, while first 10 features were selected from the NCA and NB algorithms.

### 3.5. Classification

For the current study, the support vector machine (SVM), and backpropagation multi-layer perceptron (MLP) algorithms were utilized for the classification of six basic emotion classes, including the emotions of happy, disgust, fear, surprise, anger, and sad. To obtain the multiclassification process, the one-against-one technique was used by reducing the multi-classification process to multiple binary classifications between two pairs of classes. Therefore, 15 binary classification models were utilized for each multiclassification. However, to ensure the data of each class were free of noise, the outlier process was performed before the classification by calculating the mean and standard deviation of each class, and instances with more than three standard deviations far from the mean were excluded. More importantly, to preserve balance criteria in binary classification, the down sampling technique was performed. Moreover, a 10-fold cross validation technique was utilized for the validation process. The selected kernel for the SVM classifier was the radial basis function, with an epsilon equal to 0.001, while the configuration of MLP was as follows: the learning rate was equal to 0.01, the number of epochs was 500, the backpropagation rate was 0.2, and the number of hidden layers equaled 6.

## 4. Experimental Analysis and Discussion

**Figure 4:**
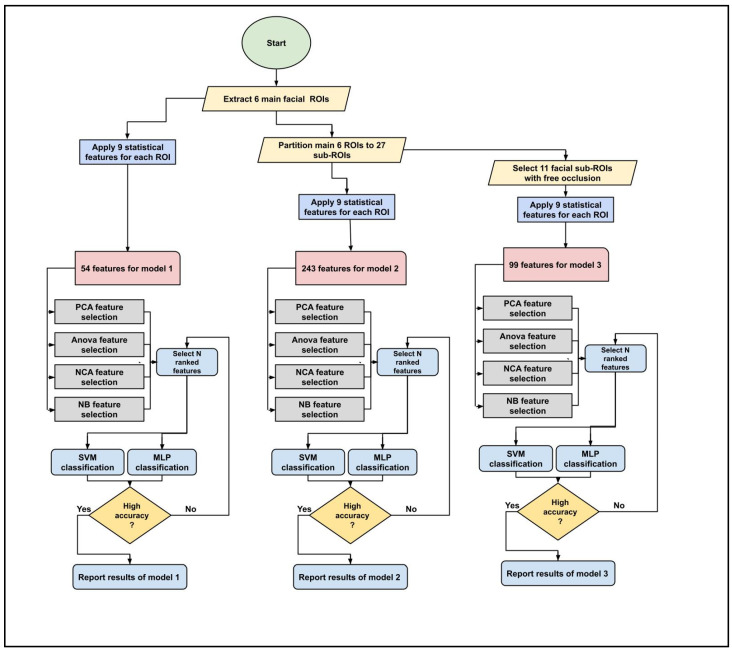
Classification process flow with multiple feature selection and classification algorithms.

This section demonstrates the experimental results obtained from three classification models and discusses the reported results based on the study’s objectives. For each classification model, the process flow was obtained as demonstrated in Figure 4. To evaluate the performance of the proposed models, three statistical analysis methods were obtained, including the precision, F1 score, and Kappa analysis.

### 4.1. Affective State Recognition Based on Six Main Facial ROIs

The goal of the proposed classification model was to explore the efficiency of six main facial ROIs to classify six basic emotions. Table 1 outlines the mean accuracy results reported from the SVM and MLP classification algorithms with PCA, ANOVA, NCA, and NB feature selectors. As shown in Table 1, the highest mean accuracy results were reported in the sad class, at 98.5%, for SVM-NCA, and the second highest mean accuracy results, at 98.4%, were reported in the sad class for SVM-NB. Moreover, the lowest mean accuracy result was reported to be 62.9% in the fear class from MLP-PCA. Furthermore, Table 2 demonstrates that SVM-NCA reported 95.3%, which is the highest overall mean recognition accuracy result, followed by MLP-NB, which reported 92.1%. The minimum overall mean accuracy results were reported at 73.8% for SVM-PCA. For the evaluation process, Table 3 outlines three statistical evaluation methods, the precision, F1 score, and Kappa, which were reported to be highest from the first two classification algorithms.

### 4.2. Affective State Recognition Based on 27 Sub-Facial ROIs

The second classification model in this study explored the efficiency of decomposing 6 main facial ROIs into 27 sub-ROIs with the implementation of 9 statistical features for each sub-ROI. Table 3 outlines the mean accuracy results reported from the proposed method. The highest mean accuracy was reported, at 97.6%, in the disgust class obtained from the MLP-NB classification, and the second highest accuracy result was reported, at 97.3%, in the disgust class from MLP-NCA. The lowest mean accuracy result was reported, at 54.1%, in the anger class from SVM-PCA. The highest overall mean accuracy results were reported to be 95.2% from SVM-NB and 95.1% from SVM-NCA. The overall lowest mean accuracy result reported was 61.6% in SVM-PCA. Table 4 demonstrates statistical evaluation methods related to a higher classification with the feature selection method used in this model.

### 4.3. Affective State Recognition Based on Selective 11 Sub-Facial ROIs

The third proposed model for the current study relied on the selection of 11 sub-ROIs from 27 sub-ROIs; the goal of this proposed model was to explore the efficiency of the selected sub-ROIs with free facial hair to avoid thermal occlusion. As demonstrated in Figure 3c, R14 and R17 are the sub-ROIs related to the upper left and right cheeks selected to avoid the existence of hair from a beard. Furthermore, the selected lower sub-ROIs in the forehead are represented by R10 to R12 in Figure 3c, also to avoid patches of forehead that could be covered by hair (bangs). Moreover, only upper and lower lips, represented by R22 to R25, were selected from the mouth region to avoid subregions, which may include facial hair such as mustaches and a beard. However, Table 5 demonstrates the mean accuracy results reported from SVM and MLP classification algorithms and PCA, ANOVA, NCA, and NB feature selectors. The disgust class reported the highest mean recognition accuracy of 96.7% in MLP-NCA and 96.1 in SVM-NCA. Surprisingly, the lowest class was reported to be 42.2% from SVM-ANOVA. Moreover, MLP-NCA reported the highest overall recognition accuracy result of 93.4%, followed by SVM-NB, which reported, at 92.8%, the second highest recognition accuracy. SVM-PCA reported a 51.5% overall lower recognition accuracy. Table 6 demonstrates the statistical evaluation method reported from the classification of the human affective state based on the selected 11 sub-ROIs and SVM and MLP classifiers with NCA and NB feature selection algorithms.

### 4.4. Comparative Study

**Figure 5:**
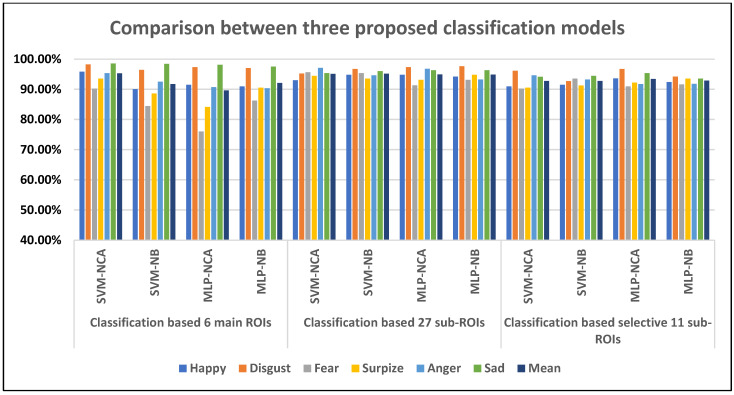
Comparison between three proposed classification models.

The highest results from the previous proposed classification models have been reported by two feature selection algorithms, namely, NCA and NB, while the lowest outcomes were identified by PCA and ANOVA. This finding may refer to the significance of feature selection algorithms in the classification process. Moreover, as aforementioned, the number of selected features could play a significant role in the classification results. In the current study, 50 higher ranked features were opted from PCA and ANOVA, while 10 higher ranked features were selected from NCA and NB. The reason for selecting 50 features from PCA and ANOVA relied on experimental trails to achieve higher accuracy results. More importantly, Figure 5 documents a comparison between the results reported from three proposed models. As shown in Figure 5, the highest overall mean accuracy result in model one was 95.3%, reported from the SVM-NCA classifier, and the highest overall mean accuracy result in model two was 95.2%, reported from SVM-NB classifiers, while the highest overall mean accuracy result in model three was 93.4%, reported from MLP-NCA. However, the results from the three proposed models appeared to be nearly identical, which means that the proposed method of decomposing main facial patches into sub-regions could help the researcher to avoid occluded facial regions in thermal images, such as eyeglasses and facial hair. Furthermore, the results of this study show that, despite using a small number of facial ROIs, the classification performance is still promising. More importantly, the current study’s findings show that increasing the number of features does not always result in a higher accuracy, while few robust features could report more significant impact.

After a comparison has been made between three proposed models, it is important to compare the proposed models with those conducted in the literature. Therefore, the current study selected models from other studies which selected their data from the USTC-NVIE [27] database. Table 7 demonstrates the comparative results obtained from the current study with other methods from the literature based on the UTSC-NVIE database. As shown in Table 7, the results reported from the current study outperform the results reported from other studies, except for the study in [41], which reported higher mean accuracy results. The reason for this could refer to the type of features that were used.

This study also validated its findings by comparing them to previous studies based on visual facial images. Table 8 outlines the comparison based on visual images and shows that the mean accuracy results reported from the current study are competitive with other results.

## 5. Conclusions

This paper proposed three modalities for spontaneous affective state recognition based on facial thermal images with eyeglass and facial hair occlusion. The main objective of this paper was to explore the facial sub-regions which were free of eyeglass and hair occlusion and more efficient in exploring the human affective state. The three proposed models were dependent on each other. The first model focused on the classification of affective states based on six main facial ROIs, and the eyeglass location was excluded. The second model decomposed the 6 main ROIs into 27 sub-ROIs to explore the efficiency of sub-facial regions in the classification of affective states. The third model in this paper selected 11 sub-ROIs from 27 ROIs to explore the ability of avoiding facial hair regions in thermal images. In comparison to previous studies, the results reported from the three proposed models demonstrate a higher mean accuracy: 95.3%, 95.2%, and 92.8% for models one, two, and three, respectively. Furthermore, the results of this study show the importance of feature selection techniques in improving classification accuracy. However, in future studies, we will focus on the automatic extraction of ROIs and employ deep learning algorithms to improve the recognition of human affective states.

## Figures and Tables

**Table 1:** Mean accuracy results for affective state recognition based on six main ROIs reported from the implementation of SVM and MLP classifiers with PCA, ANOVA, NCA, and NB feature selection algorithms.

Classifier/Feature Selector	Happy	Disgust	Fear	Surprise	Anger	Sad	Mean
SVM-PCA	74.6%	72.2%	74.2%	70.2%	71.0%	80.8%	73.8%
SVM-ANOVA	76.7%	82.5%	72.4%	68.6%	77.4%	86.4%	77.3%
SVM-NCA	95.8%	98.2%	90.2%	93.5%	95.3%	98.5%	95.3%
SVM-NB	90.0%	96.4%	84.4%	88.6%	92.5%	98.4%	91.7%
MLP-PCA	84.9%	88.2%	62.9%	76.1%	82.8%	80.2%	79.2%
MLP-ANOVA	75.5%	92.1%	70.2%	75.3%	79.9%	88.9%	80.3%
MLP-NCA	91.5%	97.3%	76.0%	84.1%	90.7%	98.1%	89.6%
MLP-NB	90.9%	97.0%	86.2%	90.5%	90.3%	97.5%	92.1%
Mean	88.1%	94.9%	78.3%	84.7%	88.6%	93.6%	88.0%

**Table 2:** Precision, F1 score, and Kappa statistical evaluation method reported from classification human affective state recognition based on six main ROIs and SVM, MLP classifiers with NCA and NB feature selection algorithms.

Classifier/Feature Selector	Evaluation	Happy	Disgust	Fear	Surprise	Anger	Sad	Mean
SVM-NCA	Precision	0.94	0.96	0.94	0.96	0.93	0.99	0.95
	F1 score	0.95	0.97	0.92	0.95	0.94	0.99	0.95
	Kappa	0.64	0.64	0.71	0.67	0.70	0.65	0.67
SVM-NB	Precision	0.92	0.90	0.89	0.89	0.92	1.00	0.92
	F1 score	0.91	0.93	0.87	0.89	0.92	0.99	0.92
	Kappa	0.65	0.63	0.72	0.68	0.70	0.65	0.67
MLP-NCA	Precision	0.89	0.85	0.93	0.97	0.89	0.90	0.91
	F1 score	0.90	0.91	0.84	0.90	0.90	0.94	0.90
	Kappa	0.64	0.62	0.74	0.69	0.70	0.64	0.64
MLP-NB	Precision	0.91	0.91	0.97	0.95	0.90	0.92	0.93
	F1 score	0.91	0.94	0.91	0.92	0.90	0.94	0.92
	Kappa	0.65	0.63	0.72	0.68	0.70	0.64	0.67

**Table 3:** Mean accuracy results for affective state recognition based on 27 sub-ROIs reported from implementation of SVM and MLP classifiers with PCA, ANOVA, NCA, and NB feature selection algorithms.

Classifier/Feature Selector	Happy	Disgust	Fear	Surprise	Anger	Sad	Mean
SVM-PCA	66.4%	55.5%	74.2%	59.8%	54.1%	59.6%	61.6%
SVM-ANOVA	66.4%	63.3%	69.5%	57.8%	55.6%	78.9%	65.2%
SVM-NCA	93.0%	95.2%	95.6%	94.4%	97.1%	95.3%	95.1%
SVM-NB	94.8%	96.7%	95.3%	93.5%	94.6%	96.0%	95.2%
MLP-PCA	66.7%	76.1%	58.5%	64.7%	82.1%	79.1%	71.2%
MLP-ANOVA	70.0%	66.7%	65.1%	61.1%	69.1%	77.0%	68.2%
MLP-NCA	94.8%	97.3%	91.3%	93.1%	96.8%	96.3%	94.9%
MLP-NB	94.2%	97.6%	93.1%	94.8%	93.2%	96.3%	94.9%
Mean	80.8%	80.7%	80.8%	77.6%	80.1%	84.6%	80.8%

**Table 4:** Precision, F1 score, and Kappa statistical evaluation method reported from classification human affective state recognition based on 27 sub-ROIs and SVM and MLP classifiers with NCA and NB feature selection algorithms.

Classifier/Feature Selector	Evaluation	Happy	Disgust	Fear	Surprise	Anger	Sad	Overall
SVM-NCA	Precision	0.96	0.94	0.95	0.93	0.96	0.96	0.95
	F1 score	0.94	0.95	0.95	0.94	0.97	0.96	0.95
	Kappa	0.65	0.64	0.70	0.67	0.70	0.66	0.67
SVM-NB	Precision	0.94	0.97	0.92	0.93	0.96	0.98	0.95
	F1 score	0.94	0.97	0.94	0.93	0.96	0.97	0.95
	Kappa	0.65	0.65	0.70	0.67	0.70	0.65	0.67
MLP-NCA	Precision	0.93	0.93	0.98	0.95	0.96	0.96	0.95
	F1 score	0.94	0.95	0.94	0.94	0.96	0.96	0.95
	Kappa	0.64	0.64	0.71	0.68	0.70	0.65	0.67
MLP-NB	Precision	0.93	0.93	0.98	0.95	0.94	0.97	0.95
	F1 score	0.94	0.95	0.95	0.95	0.94	0.97	0.95
	Kappa	0.65	0.64	0.71	0.67	0.70	0.65	0.67

**Table 5:** Mean accuracy results for affective state recognition based on selective 11 sub-ROIs reported from implementation of SVM and MLP classifiers with PCA, ANOVA, NCA, and NB feature selection algorithms.

Classifier/Feature Selector	Happy	Disgust	Fear	Surprise	Anger	Sad	Mean
SVM-PCA	56.4%	49.4%	48.4%	55.9%	47.0%	51.9%	51.5%
SVM-ANOVA	63.0%	55.9%	61.6%	42.2%	55.9%	79.2%	59.6%
SVM-NCA	90.9%	96.1%	90.2%	90.5%	94.6%	94.1%	92.7%
SVM-NB	91.5%	92.7%	93.5%	91.2%	93.2%	94.4%	92.8%
MLP-PCA	48.8%	50.6%	50.5%	52.9%	55.9%	50.6%	51.6%
MLP-ANOVA	68.8%	67.9%	62.8%	59.5%	59.9%	86.0%	67.5%
MLP-NCA	93.6%	96.7%	90.9%	92.2%	91.7%	95.3%	93.4%
MLP-NB	92.4%	94.2%	91.6%	93.5%	91.8%	93.5%	92.8%
Mean	75.7%	75.4%	73.7%	72.2%	73.7%	80.6%	75.2%

**Table 6:** Precision, F1 score, and Kappa statistical evaluation method reported from classification human affective state recognition based on selective 11 sub-ROIs and SVM and MLP classifiers with NCA and NB feature selection algorithms.

Classifier/Feature Selector	Evaluation	Happy	Disgust	Fear	Surprise	Anger	Sad	Overall
SVM-NCA	Precision	0.93	0.92	0.95	0.91	0.92	0.94	0.93
	F1 score	0.92	0.94	0.92	0.91	0.93	0.94	0.93
	Kappa	0.65	0.64	0.71	0.67	0.70	0.65	0.67
SVM-NB	Precision	0.93	0.92	0.93	0.92	0.94	0.93	0.93
	F1 score	0.92	0.92	0.93	0.92	0.93	0.94	0.93
	Kappa	0.65	0.65	0.70	0.67	0.70	0.65	0.67
MLP-NCA	Precision	0.93	0.93	1	0.93	0.91	0.92	0.94
	F1 score	0.93	0.95	0.95	0.93	0.91	0.94	0.94
	Kappa	0.65	0.64	0.72	0.67	0.70	0.65	0.67
MLP-NB	Precision	0.93	0.92	0.96	0.91	0.95	0.92	0.93
	F1 score	0.93	0.93	0.94	0.92	0.93	0.93	0.93
	Kappa	0.65	0.64	0.71	0.67	0.71	0.65	0.67

**Table 7:** Comparative results with other literature studies based on USTC-NVIE database.

Methods	Features	ROIs	Classifier	Accuracy	Precession	F1 Score	Kappa
[22]	LBP	Selected facial sub-ROIs	Multiclass SVM	78.16%	78.3%	0.774	-
[51]	Statistical features	100 sub-ROIs without facial area	SVM	76.45%	76.6%	0.753	0.637
[41]	Transfer Learning	Hybrid approach	Multiclass SVM	99.3%	98.5%	0.984	0.93
[6]	Sparce coded filter	Selected facial sub-ROIs	SVM	66.1%	67.1%	0.663	0.179
Our Proposed methods	Statistical features	6 main facial ROIs	SVM-NCA	95.3%	95%	0.95	0.67
	Statistical features	27 facial sub-ROIs	SVM-NB	95.2%	95%	0.95	0.67
	Statistical features	Selective 11 facial sub-ROIs	SVM-NB	92.8%	93%	0.93	0.67

**Table 8:** Comparative results with other studies in the literature based on visual facial images dataset.

Methods	Datasets	Features	Classifier	Accuracy
Proposed model one	USTC-NVIE	Statistical features	SVM-NCA	95.3%
Proposed model two	USTC-NVIE	Statistical features	SVM-NB	95.2%
Proposed model three	USTC-NVIE	Statistical features	SVM-NB	92.8
[52]	JAFFE	Haar, Gabor, LBP	SVM, KNN	89.5%
[53]	JAFFE	facial landmarks, and center of gravity	SVM	91.9%
[54]	JAFFE	DBN	ANN	90.95%
[55]	KDEF	Transfer learning	CNN	98.78%
	JAFFE	Transfer learning	CNN	99.52%

## Data Availability

Data sharing not applicable.
